# 3D X-ray tomographic analysis reveals how coesite is preserved in Muong Nong-type tektites

**DOI:** 10.1038/s41598-020-76727-6

**Published:** 2020-11-26

**Authors:** Matteo Masotta, Stefano Peres, Luigi Folco, Lucia Mancini, Pierre Rochette, Billy P. Glass, Fabrizio Campanale, Nicolas Gueninchault, Francesco Radica, Sounthone Singsoupho, Enrique Navarro

**Affiliations:** 1 Dipartimento di Scienze della Terra, Università di Pisa, Pisa, Italy; 2 CISUP, Centro per l’Integrazione della Strumentazione dell’Università di Pisa, Pisa, Italy; 3 Department of Lithospheric Research, University of Vienna, Wien, Austria; 4 Elettra-Sincrotrone Trieste SCpA, Basovizza, Trieste, Italy; 5 LINXS-Lund Institute for Advanced Neutron and X-Ray Science, Lund, Sweden; 6 CEREGE, Aix-Marseille University, CNRS, INRA, IRD, Aix-en-Provence, France; 7 Department of Earth Sciences, University of Delaware, Newark, DE USA; 8 Center for Nanotechnology Innovation@NEST, Istituto Italiano di Tecnologia (IIT), Pisa, Italy; 9 Zeiss Research Microscopy Solutions, Carl Zeiss SAS, Marly-le-Roi, France; 10 Dipartimento di Scienze, Università degli Studi Roma Tre, Roma, Italy; 11 Department of Physics, Faculty of Natural Sciences, National University of Laos, Vientiane, Laos; 12 Instituto di Geologia y Geofisica CIGEO, Universidad Nacional Autónoma de Nicaragua, Managua, Nicaragua

**Keywords:** Meteoritics, Mineralogy

## Abstract

Muong Nong-type (MN) tektites are a layered type of tektite associated to the Australasian strewn field, the youngest (790 kyr) and largest on Earth. In some MN tektites, coesite is observed in association with relict quartz and silica glass within inclusions surrounded by a froth layer. The formation of coesite-bearing frothy inclusions is here investigated through a 3D textural multiscale analysis of the vesicles contained in a MN tektite sample, combined with compositional and spectroscopic data. The vesicle size distribution testifies to a post-shock decompression that induced melting and extensive vesiculation in the tektite melt. Compared to free vesicles, nucleated homogeneously in the tektite melt, froth vesicles nucleated heterogeneously on relict quartz surfaces at the margins of coesite-bearing inclusions. The rapid detachment of the froth vesicles and prompt reactivation of the nucleation site favoured the packing of vesicles and the formation of the froth structure. Vesicle relaxation time scales suggest that the vesiculation process lasted few seconds. The formation of the froth layer was instrumental for the preservation of coesite, promoting quenching of the inclusion core through the subtraction of heat during froth expansion, thereby physically insulating the inclusion until the final quench of the tektite melt.

## Introduction

Coesite is a high-pressure polymorph of SiO_2_ and a diagnostic feature of shock metamorphism associated with impact cratering on quartz-bearing target rocks^1^. The preservation of coesite as a metastable phase in shocked rocks that experienced peak pressures and temperatures much beyond its stability field (i.e., pressures of 3–10 GPa and temperatures below 2700 °C) represents a controversial issue^2^. For this reason, different mechanisms of coesite formation have been proposed, including coesite crystallization from an impact silica melt during shock unloading^3–5^, subsolidus nucleation from highly densified diaplectic silica glass^6^ and direct subsolidus quartz–coesite transition upon shockwave reverberation^2,7^. Regardless of the formation mechanism, the kinetic of the coesite → quartz transformation determines the possibility of coesite survival in a transient high-pressure and high-temperature regime (e.g.^8^). Hence, in order to explain the survival of coesite in impact rocks, a combined examination of the textural features and of the physical conditions of the shock is required.

In the Australasian tektite strewn field, coesite was found as microcrystalline grains in partly melted mineral inclusions within impact melt glasses (namely tektites^9,10^) and in shocked ejecta found in the Australasian microtektite layer^7,11^. Muong Nong (MN) tektites constitute one of the three tektite types associated with the Australasian strewn field and the only one where coesite was occasionally found^9,10^. Compared to splashform and ablated types, MN tektites typically occur as small fragments (with the exception of a few very large samples, weighting some tens of kg) with no signs of ablation. They are characterized by a heterogeneous chemical composition and exhibit a characteristic layering, for which they are also known as layered tektites^12^. Moreover, they display a higher water content (up to 300 ppm) as compared to splashform tektites (up to 209 ppm)^13,14^ and microtektites collected in Antarctica (< 20–206 ppm)^15^ and a lower concentration of ^10^Be as compared to ablated tektites from Australia^16^ and microtektites from Antarctica^17^. These geochemical and isotopic data indicate a deeper origin in the target rock of MN tektites and a consequently lower ejection velocity, which suggests that they are closer to the source crater than tektites from the rest of the strewn field outside of Indochina^18^. An impact location in Indochina is actually consistent with the geographic distribution and other petrographic and geochemical trends in both tektites and microtektites^19–24^ and, recently, a gravity anomaly observed in Laos was proposed as geophysical evidence of a crater buried under younger volcanic rocks of the Bolaven volcanic field^25^.

A detailed petrographic and chemical investigation of the MN tektite examined in this study (sample MP26 from Muang Phin, Laos) has been presented by Glass et al.^10^. Characteristic features of sample MP26 are: i) the layering of the vesicle-bearing silicate melt with slightly variable composition, ii) the occurrence of inclusions constituted by silica glass (hereafter referred to as lechatelierite inclusions) and iii) the presence of white opaque inclusions constituted by a densely vesiculated (froth) layer of SiO_2_-rich melt enclosing a core composed by a mixture of coesite, relict quartz and silica glass (hereafter referred to as coesite-bearing frothy inclusions). Glass et al.^10^ proposed that the presence of the froth layer in the coesite-bearing inclusions was fundamental for the survival of the coesite, as it acted as a heat sink and physical barrier during the solidification of the tektite. Nonetheless, the mechanism of formation of such an uncommon petrographic feature and the source of the volatile phase responsible of the vesiculation of MN tektite remain unsolved. These are crucial factors in defining the physical conditions for the formation of MN tektites and, more specifically, in understanding the entire process of coesite formation and survival in impact melt rocks.

In order to address these problems, we investigated the mechanisms and time scales of vesicle formation, in the general frame of tektite formation process. For this purpose, we performed a multiscale, three-dimensional (3D) characterization of the vesicles contained in MP26 by combining laboratory-based and synchrotron radiation (SR) phase-contrast X-ray computed microtomography (µCT) imaging techniques. Laboratory-based CT measurements have been performed using a custom-developed microfocus system (MCT) and a commercial 3D X-ray microscope (XRM). By integrating the 3D textural data with micro-chemical and spectroscopic data (Fourier-transform infrared spectroscopy, FTIR), we reconstructed the mechanisms of vesiculation and their time scales during the formation of the MN tektite, corroborating the hypothesis that the development of froth structures constitutes an effective process for the preservation of coesite and other key shock-metamorphic features in impact melt rocks.

## Results

### 2D textural and chemical analysis

**Figure 1:**
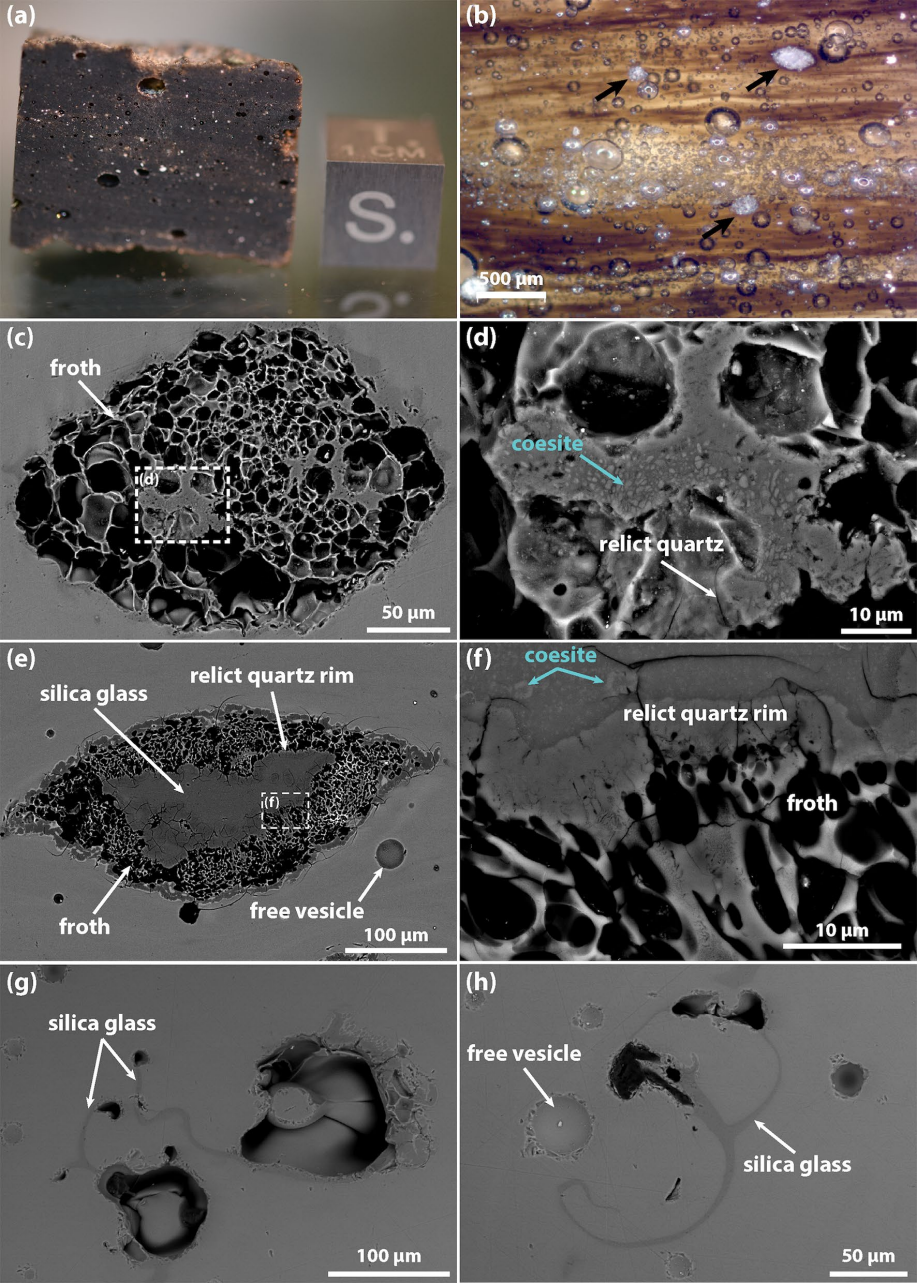
Sample MP26 **(a)** and optical microscope image of a thin slab of MP26, the location of frothy inclusions is indicated by the arrows **(b)**. Backscattered electron images of a sub-spherical coesite-bearing inclusion, characterized by coesite microcrystals and relict quartz crystals **(c,d)**. Backscattered electron images of an elongated coesite bearing inclusion (from Glass et al.^10^), characterized by a relict quartz rim and froth vesicles that are increasingly larger outward from the inclusion **(e,f)**. Backscattered electron images of two lechatelierite inclusions **(g,h)**.

Optical microscope images of MP26 show a characteristic layering, made evident by the brown shades of the glassy bands and by the common orientation of larger (> 500 µm; referred to the length of the major axis), elongated vesicles (Fig. 1a). The distribution of the vesicles on a polished surface is rather inhomogeneous, with the higher concentration of vesicles observed in planes parallel to the layering of the tektite, corresponding to certain glassy bands and regardless of the colour (Fig. 1b). The chemical composition of the tektite glass is very similar to that of a volcanic glass (namely a rhyolite) or a dominantly quartz-feldspatic lithology (Table S1). The colour shades of the glass bands correspond to variable intensities in the backscattered electron (BSE) images and are related to small compositional heterogeneities of the tektite. Pale brown glassy bands appear darker in the BSE images and display a small enrichment in SiO_2_ (up to 5 wt.% higher) as compared to the dark brown bands, which appear brighter in the BSE images as result of slightly higher FeO, MgO and CaO contents (up to 1 wt.% for each oxide). Within this variability, the major element composition is comparable with chemical data reported in literature^10,18,26^ for MN tektites (including MP26).

Observed at the microscale, the vesicles occur either as individual vesicles isolated within the tektite glass (free vesicles) or as compact vesicle aggregates in the froth surrounding the coesite-bearing frothy inclusions (froth vesicles). The free vesicles are normally larger than the froth vesicles and range in size from 10 to 2000 µm (with the exception of a few very large vesicles with length of the major axis > 2000 µm). Their cross-sectional shape is mostly circular, although larger (> 500 µm) vesicles appear elliptical and with a preferential elongation parallel to the layering of the tektite. In contrast, the smaller (5–50 µm) froth vesicles exhibit less spherical shapes and overall increase in size from the contact with the inclusion towards the tektite melt (Fig. 1c).

Inclusions are essentially of two types, coesite-bearing frothy inclusions (Fig. 1c–f) and lechatelierite inclusions (Fig. 1g,h). Coesite-bearing frothy inclusions are generally larger (200–600 µm) and more abundant than lechatelierite ones (100–300 µm). Glass et al.^10^ described the texture of these inclusions and reported a number of ~ 14 inclusions per cm^−2^. The core of the inclusions is generally characterized by silica glass, coexisting with microcrystalline (< 2 µm) aggregates of coesite and relict quartz grains, whilst towards the edge, the relict quartz grains are often arranged to form a rim that separates the core of the inclusion from the outer, up to 120 µm thick, froth layer (Fig. 1e,f). In many inclusions, the relict quartz rim is discontinuous and exhibits gaps where the froth layer appears less developed and the inclusion core is in contact with the outer tektite melt (see Fig. 2 of Glass et al.^10^). Compared to coesite-bearing frothy inclusions, lechatelierite inclusions are characterized by silica glass and large (> 20 µm), convoluted vesicles (Fig. 1 g,h). Small (5–10 µm) and rare inclusions of Fe–Ni–S alloys have been also observed, but not further investigated (cfr. ^27^). As already observed for the vesicles, a spatial relationship exists between the tektite layering and the shape and distribution of coesite-bearing frothy inclusions. Both the elongation and concentration of the inclusions increase in the layers containing the higher concentration of vesicles.

**Figure 2:**
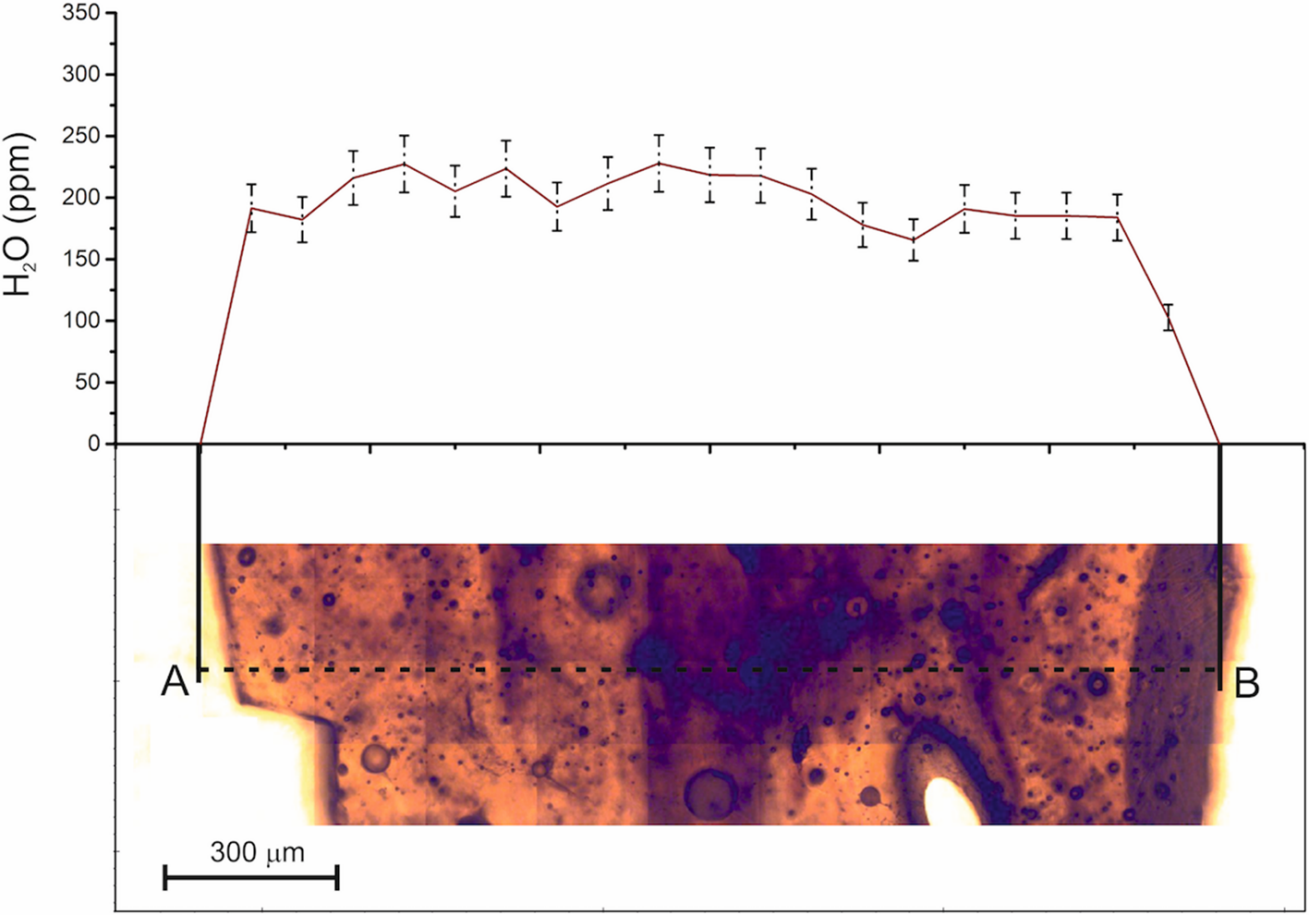
Fourier-transform infrared spectroscopy (FTIR) transect obtained on a 285 µm slab of MP26 showing the intensity of H_2_O absorbance in the different bands. The error bars indicate the uncertainty in the H_2_O measurement in each point of the transect.

The H_2_O concentration in MP26, determined using FTIR spectroscopy along a transect perpendicular to the layering, ranges from a minimum of 166 ppm to a maximum of 228 ppm, with an average value of 200 ± 18 ppm (Fig. 2). No correlation is observed between the H_2_O concentration and the colour/composition of the glass bands of the tektite. Our measurements are consistent with previous determinations of H_2_O content in MN tektites^13^ and confirm that MP26 is slightly enriched in H_2_O compared to other Australasian distal ejecta, such as splashform tektites and the microtektites found in the deep ocean sediments and in the transantarctic mountains (< 20–246 ppm)^13,15^.

### 3D morphology and distribution of vesicles and coesite-bearing frothy inclusions

A multiscale 3D textural analysis was performed on different volumes of interest (*VOIs*) by combining different X-ray µCT instruments, varying the voxel size from 12.48 to 0.90 µm (Table 1). Due to the heterogeneity of MN tektite, a minimum representative elementary volume (*REV*) of 60 mm^3^ was determined. Consistent results of bulk vesicularity (*ϕ*) of 4.7–4.9 vol.% are obtained only for *VOIs* larger than the *REV*, whereas reliable vesicle number density (*VND*) values in the order of 10^3^–10^4^ mm^-3^ are obtained only in the analysis of smaller *VOIs* at higher spatial resolution (allowing to detect and analyse vesicles with diameter as small as 3 µm).

**Table 1:** Summary of 3D textural analysis of VOIs extracted from XRM, MCT and SR-µCT data on MP26.

Sample	Method	Isotropic voxel size (µm)	Cut-off (µm)	*VOI *(mm^3^)	*Φ *(vol. %)	*VND *(mm^−3^)
MP26a	XRM	12.48	15	482	4.7	1.3 × 10^2^
MP26a	XRM	4.24	10	60	4.8	2.9 × 10^2^
MP26a	XRM	1.09	3	0.82	8.9	5.2 × 10^3^
MP26a	MCT	5.00	10	125	4.9	2.6 × 10^2^
MP26b	SR-µCT	2.50	5	20.36	8.8	4.3 × 10^3^
MP26b^a^	SR-µCT	2.50	5	20.36	0.4	9.8
MP26c	SR-µCT	0.90	3	0.67	2.2	4.3 × 10^3^

^a^Segmentation of coesite-bearing frothy inclusions in the same VOI used for MP26b (row above).

**Figure 3:**
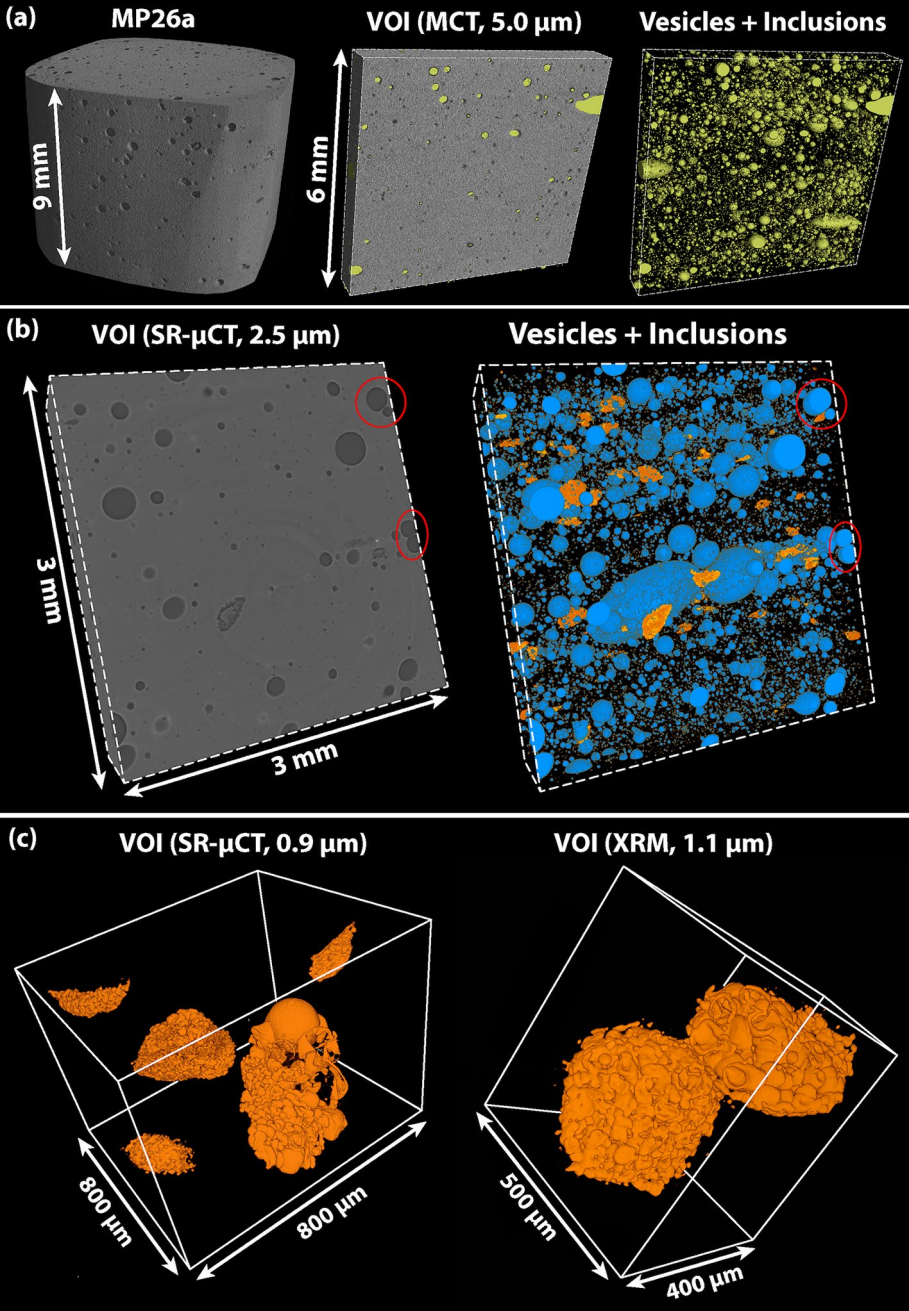
Volume rendering of MP26a obtained by MCT data reconstructed with an isotropic voxel size of 5.0 µm and isosurface rendering after segmentation of all voids **(a).** Volume rendering of MP26b obtained using SR-µCT data reconstructed with an isotropic voxel size of 2.5 µm and isosurface rendering after segmentation of vesicles (coloured in blue) and inclusions (coloured in orange); red circles indicate free vesicles quenched before coalescence **(b)**. Isosurface rendering of coesite-bearing frothy inclusions obtained using SR-µCT data reconstructed with an isotropic voxel size of 0.9 µm and XRM data reconstructed with an isotropic voxel size of 1.09 µm **(c)**.

As already observed by 2D microscopy analyses, the 3D morphology of the free vesicles ranges from nearly spherical to ellipsoidal, with the larger (length of the major axis > 200 µm) vesicles showing the higher deviation from the spherical shape and a common orientation parallel to the layering of the tektite (Fig. 3a; Video SV1). The 3D imaging characterization of the investigated *VOIs* reveals regions with different density of vesicles and inclusions that correspond to different layers of the tektite (Fig. 3b). The coesite-bearing frothy inclusions can be identified as individual objects, as they appear enclosed by the densely-packed froth vesicles, whose shapes range from spherical to tubular and belt-shaped (Fig. 3c; Fig. S4 and Video SV2).

**Figure 4:**
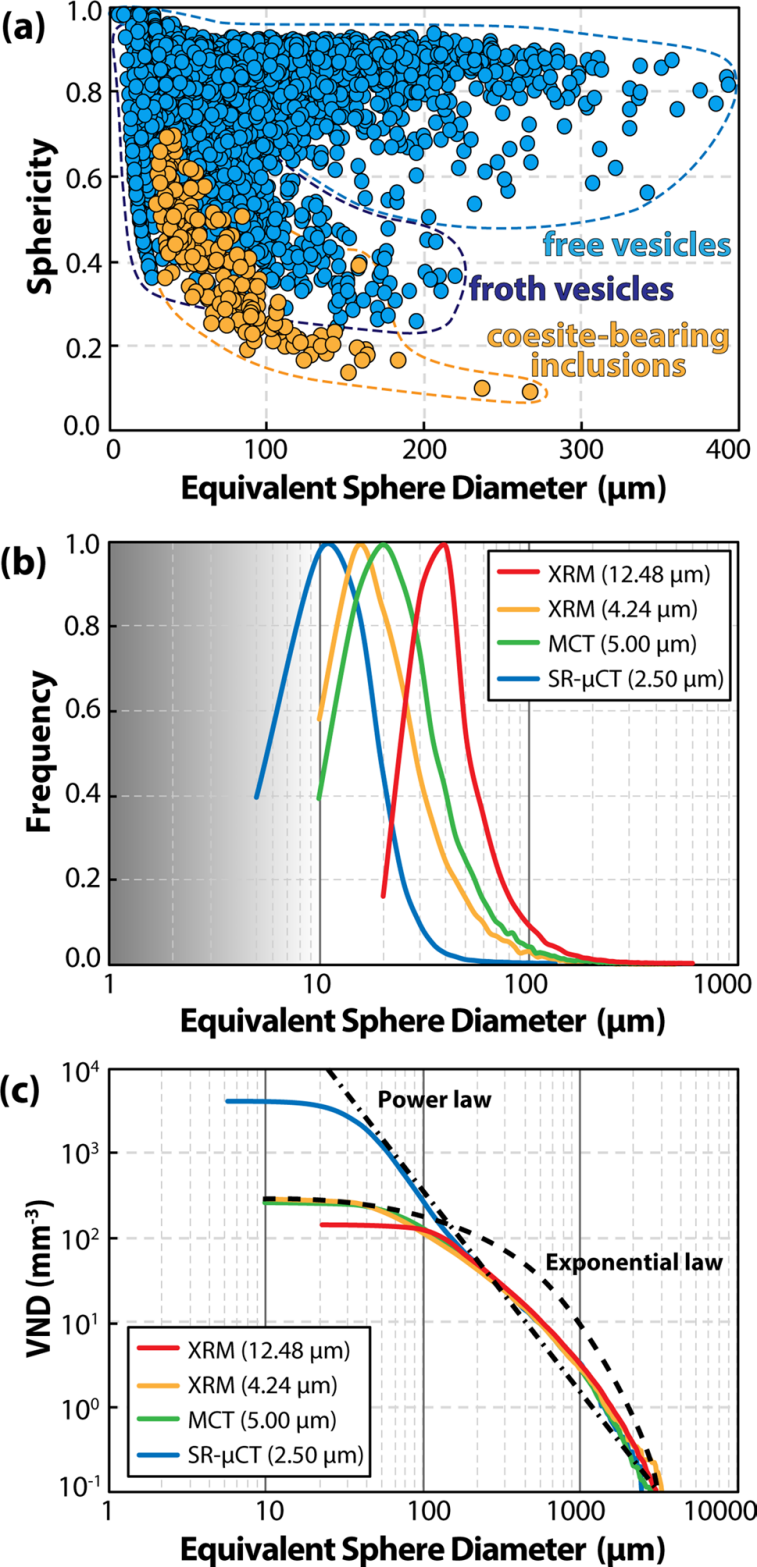
Results of 3D analysis of textural parameters for different volumes of MP26 analysed at different voxel resolutions. The sphericity of both free and froth vesicles (blue circles) and of coesite-bearing frothy inclusions (orange circles) is plotted versus the diameter of the sphere with equivalent volume; note that the froth vesicles and free vesicles are distinguished based on their different trends and that the division between the two trends (blue dashed lines) is drawn by eye **(a)**. Vesicle size distribution (VSD) frequency histogram (x axis in logarithmic scale) **(b)** and cumulative plot indicating the vesicle number density (VND) of each vesicle size class **(c)**.

The 3D analysis of textural parameters obtained from all the analytical datasets (XRM, MCT and SR-µCT) consistently confirms the decrease of the sphericity with increasing size of both free and froth vesicles. Note that the two types of vesicles form two different trends in Fig. 4a, with froth vesicles having much lower sphericity than free vesicles, due to their irregular morphology (Fig. 3c). A similar trend is observed for coesite-bearing frothy inclusions, whose lower sphericity is due to the irregular shape of the core and enhanced by the presence of the froth layer (Fig. 4a; Fig. S4). The vesicle size distribution (*VSD*) curves for the vesicles display continuous trends of decreasing abundance with increasing size, with median values in the classes with equivalent sphere diameter of 10–15 µm (SR-µCT data with isotropic voxel size of 0.90 and 2.50 µm and XRM data with isotropic voxel size of 1.06 µm) and 15–20 µm (XRM and MCT data with isotropic voxel size of 12.48, 5.00 and 4.24 µm) (Fig. 4b). The difference in the median value is related to the different cut-off imposed by the voxel resolution. We note that the 3D textural analyses obtained at higher voxel resolution, although calculated on *VOIs* smaller than the *REV* size, yield much higher *VND* values and appear thus more representative of the actual density of vesicles in MN tektite (Table 1). In spite of the shift towards lower median values at increasing voxel resolution, all *VSD* curves exhibit comparable positive-skews, indicating a non-linear increase in abundance of vesicles with decreasing vesicle size. In the cumulative *VSD*, the sharp increase of the smaller vesicles translates into a deviation from the exponential relation towards a power law (Fig. 4c).

**Figure 5:**
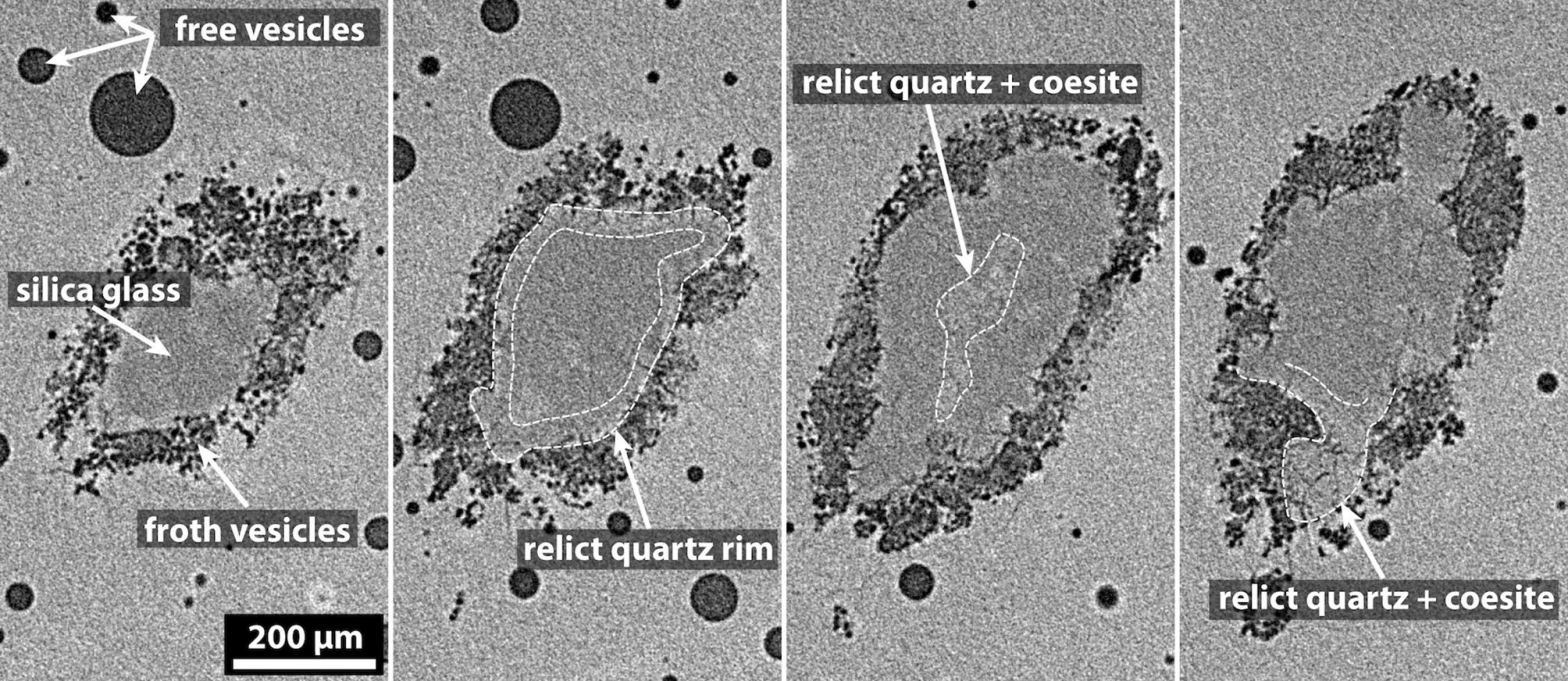
Sequence of 2D slices of a coesite-bearing frothy inclusion in MP26a obtained using XRM data reconstructed with an isotropic voxel size of 0.998 µm. The spatial distribution of vesicles, silica glass, relict quartz and coesite is inferred based on the phase contrast and indicated by the dashed lines.

The 3D characterization of the coesite-bearing frothy inclusions further confirms the main features observed in 2D, that are: (i) the range of inclusion size of 200–600 µm (length of the major axis) and elongation nearly parallel to the tektite layering (Fig. 3c; Fig. S4), (ii) the presence of a (up to) 120 µm thick froth layer enclosing iii) a core composed by a mixture of silica phases (Fig. 1c,f). The presence of a core in most of the inclusions is confirmed by the 2D slices across the inclusions, where the denser SiO_2_ phases (quartz and coesite) appear as bright spots arranged in patches or, in the case of the relict quartz rim, as a bright edge separating the inclusion core from the froth (Fig. 5). Overall, the inclusion cores have a darker appearance compared to the tektite glass, testifying to a lower bulk density of the mixture of the SiO_2_ phases compared to the rest of the tektite. The frothy inclusions constitute about 0.5 vol.% of MP26, with a number density of 9.8 mm^−3^ in a volume of 20.36 mm^3^ (Table 1). In this specific volume, the number of inclusions per area measured in the 2D slices ranges from 1 to 3 mm^−2^, that is roughly one order of magnitude higher than the value of 0.14 mm^−2^ estimated by Glass et al.^10^. This difference could be explained by the fact that the analysis of the frothy inclusion would require a much larger *REV* (given their low number density compared to the free vesicles) and that not all the identified frothy inclusions are actually coesite-bearing or contain a core.

## Discussion

### Impact origin of MN tektite

In the lack of a confirmed impact site, the presence of shocked quartz, coesite and former reidite in MN tektites has been the main evidence for the impact cratering origin^20,23,28^, in contrast with the airburst hypothesis^29^. The new textural and chemical analyses of MP26 are basically consistent with previous work indicating an impact origin of the MN tektite^10^ and point to a porous target material, most probably a fine-grained quartz-rich sedimentary deposit^20,30^, able to preserve the observed short scale (15–20 µm) compositional layering (Fig. S1).

### Volatiles in MN tektite

One of the main questions on the formation of MN tektites is related to the origin and composition of the gas phase responsible for the vesiculation of the tektite melt. Vesicles in MN tektites are composed dominantly by CO_2_, with minor amounts of O_2_, CO and noble gases^31^, whilst, surprisingly, no measurable amount of H_2_O has ever been reported^32^. Compared to other tektites from the Australasian strewn field, MN tektites are characterized by a higher gas pressure in the vesicles (up to 1/3 of atmospheric pressure^33^) and a noble gas composition enriched in Ne and Ar^34^. The gas pressure of the vesicles and the noble gas composition are normally used to estimate the height to which tektites were ejected by the impact before their quench in air^35,36^. However, given the atmospheric origin of noble gases, the formation of the vesicles in the tektite must be attributed to volatile species derived from the target rock.

If we assume that the lithology was a near-surface fine-grained quartz-rich sediment, it is highly unlikely that minor gas species other than H_2_O were present in sufficient concentration to explain the extensive vesiculation in the tektite melt, yielding to the high *VND* (> 10^3^ mm^3^) and low porosity (4.7–4.9 vol.%; Table 1). Reasonably, H_2_O must have constituted the most abundant volatile component in the target rock and have nearly completely evaporated during the impact. The exceptionally low concentration of H_2_O in all tektites is indeed one of the main arguments for the impact origin and could be explained through a mechanism of dissociation of the H_2_O molecules into their atomic components^37^. The H_2_O dissociation into H_2_ + ½ O_2_ requires a very high energy and, in experimental systems, it is generally obtained from a vapour phase at high temperatures (> > 1000 °C) produced by electric discharge^38^. For comparison with tektites, atomic bomb glasses that form under energetically comparable conditions yield comparable H_2_O concentration in the order of a few ppm^39^. Hence, assuming that a high amount of energy was available during the shock, the extensive vesiculation of MP26 can be reasonably interpreted as caused by the dissociation of the H_2_O molecules and rapid exsolution of their atomic components (mostly H_2_). In this regard, it is worth noting that the solubility of H_2_ in silicate melts can be orders of magnitude lower than that of H_2_O^40,41^, which points to a high initial supersaturation and to a consequently high nucleation. Such a mechanism of vesiculation could thus explain the presence of H_2_ as minor volatile component in a large number of tektites worldwide^32^.

Based on these considerations, as alternative to the atmospheric re-equilibration of the gas pressure in the vesicles during the ejection of the tektite^35^, the low pressure of the gas phase in the vesicles of MN tektite could be possibly explained by two concurring mechanisms: (i) bonding of H_2_ and O_2_ and further adsorption of H_2_O in the tektite glass and (ii) isochoric quench of the vesicle and eventual drop of the internal gas pressure (resulting from the gas equation of state). In this latter case, assuming an ideal behaviour of the gas phase contained in the vesicles and no exchange of mass from-toward the vesicle, the gas pressure is expected to drop to values of 10^–2^–10^–3^ bar, that are consistent with the pressure values typically measured in tektite vesicles^32^.

### Vesiculation mechanisms in the tektite melt

The presence of low-density gas vesicles in tektites suggests that the exsolution of the volatile phases initiated shortly after the shock-induced melting and terminated upon complete pressure release, before the quench of the tektite melt. Vesicle nucleation occurred both homogeneously in the tektite melt (free vesicles) and heterogeneously on the surface of relict quartz crystals (froth vesicles; Fig. 1f). The substantial difference between the two mechanisms is the supersaturation pressure (*∆P*) required to initiate nucleation, that is largely reduced in the case of the heterogeneous nucleation^42^. The supersaturation pressure is expressed as the difference between the theoretical pressure of saturation of a volatile species dissolved in the melt and the actual pressure at which the volatile phase is exsolved. The value of *∆P* generally increases with the increasing polymerization of the silicate melt and decreases with the increasing temperature^43^. In the case of MP26, the contrasting effect to nucleation due to the highly-polymerized nature of the tektite melt (SiO_2_-rich composition; Table S1) was necessarily counterbalanced by the very high temperature and the consequent reduction of surface tension.

Although it is not possible to estimate the initial condition of supersaturation (the H_2_O + CO_2_ content in the target material is unknown), a progressive increase of the *∆P* value during the decompression stage can be inferred from the analysis of the *VSD* curves (Fig. 4b,c). Any departure from a near-equilibrium degassing trend is normally expressed by non-symmetric *VSD* and by a deviation from the exponential law in the cumulative *VSD*^44,45^. Indeed, in the case of MP26, positive skews are observed in all the *VSDs* and the cumulative *VSDs* deviate from exponential to power law, suggesting an increase of the nucleation rate over time that is consistent with a non-linear pressure decrease during the post-shock decompression. At these conditions, the rapid increase of *∆P* would rather favour the nucleation of new vesicles than the diffusive growth of the existing ones, limiting the possibility for them to coalesce^46^. This is evident in large adjacent free vesicles that underwent significant deformation without disruption of the melt septa (red circles in Fig. 3b) and in the densely-packed froth vesicles surrounding the coesite-bearing inclusions (Figs. 1c and 3c).

**Figure 6:**
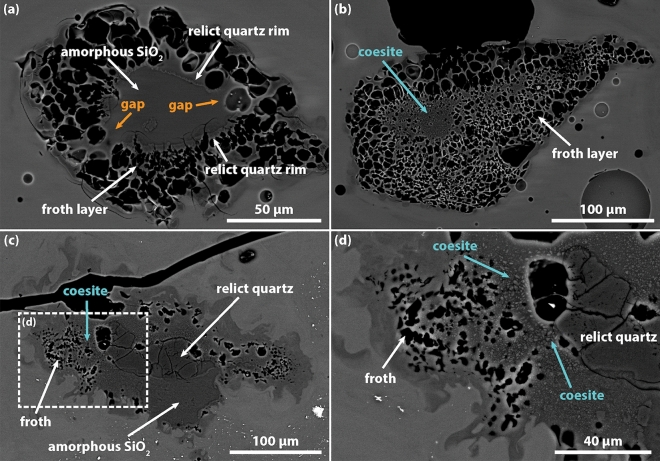
Backscattered electron images of coesite-bearing frothy inclusions from sample MN20 showing textural features comparable to MP26 **(a,b)**. Note the presence of gaps in the relict quartz rim **(a)** and the outward increasing size of the vesicle constituting the froth layer. Coesite-bearing frothy inclusions from an impact glass of Pantasma crater (sample P20-3), Nicaragua, showing textural features similar to those described for coesite-bearing frothy inclusions of MN tektites **(c,d)**.

In this frame of rapid decompression with increase of the *∆P*, the presence of the relict quartz rim surrounding the core of the inclusions was determinant for the formation of the froth layer (Fig. 1f). The surface of the relict quartz offered a preferential site for heterogeneous nucleation, by lowering the *∆P* required to initiate nucleation. On these sites, nucleation occurred earlier compared to the rest of the tektite melt and unceasingly during the decompression (or possibly during pressure reverberations associated to the pore collapse^2^). A high and constant nucleation rate on the relict quartz surface was however necessary to sustain the formation of the densely-packed vesicle structure of the froth. This was possible because of the low wetting angle of quartz, allowing for a very fast detachment of yet small vesicles from the nucleation surface and consequent reactivation of the nucleation sites^47^. The process of reactivation of the nucleation site and generation of a froth structure has been observed experimentally in silicate melts at conditions of high volatile supersaturation. For example, through µCT in situ observation of H_2_O vesiculation experiments in crystal bearing silicate melts at high temperature, Pleše et al.^48^ described froth-like textures around silicate minerals, formed in response of a rapid decrease of the wetting angle. Analogue conditions were reproduced in carbonate assimilation experiments at high temperature by Blythe et al.^49^, who concluded that the rapid removal of CO_2_ vesicle from the nucleation site was responsible for the reactivation of the nucleation site. In both types of experiments, the coalescence of adjacent vesicles induced by their growth and expansion reduced the surface available for nucleation. In the case of MP26, the rather small (5–50 µm) and homogeneous size of the froth vesicles indicates that coalescence was probably not efficient over the (very short) decompression time and that vesicle nucleation progressed unceasingly till the quench of froth structure. It must be noted, however, that few larger (> 50 µm) vesicles are sometimes observed in the outer froth layer of some coesite-bearing inclusions (Fig. 3c; Video SV2) and these may be the result of combined diffusive growth and coalescence mechanisms. We hypothesize that these vesicles are likely to be observed in inclusions presenting gaps in the relict quartz rim, thus lacking of surfaces for heterogeneous vesicle nucleation (see also Fig. 6a and further discussion below). A similar scenario may explain the lack of froth structures in lechatelierite particles and the typical occurrence of few larger vesicles, possibly resulting from the collapse of former froth vesicles following the complete melting of relict quartz (and the whole inclusion core) to form silica glass (Fig. 1g,h).

### Time scale of vesiculation and tektite formation

We argued that all vesicles in MP26 formed very rapidly during the post-shock decompression and that their preservation was possible because of the subsequent quench of the tektite melt. Hence, the time scale of vesiculation (*τ*_*ves*_) must be comprised between the time of the shock melting (*t*_*m*_) and the time of the quench (*t*_*q*_) of the tektite melt (*τ*_*ves*_* ≤ t*_*q*_* – t*_*m*_). In order to determine this time, we must consider that the tektite underwent shear deformation during the peak pressure and all free vesicles were subject to elongation, but only the smaller (< 50 µm) vesicles recovered to a fully spherical shape before the quench (Fig. 4b). The time necessary to recover the spherical shape is proportional to the size of the vesicle and the fact that the larger vesicles remained evidently ellipsoidal (e.g., very large vesicles in Fig. 3b) indicates that the time scale of vesiculation was comparable, or slightly shorter, than the time scale of vesicle relaxation (*τ*_*rel*_). Therefore, if we assume that shock decompression was nearly instantaneous and that a transient deformation of the vesicles occurred during the ejection of the tektite, we can reasonably compare the vesiculation time scale (*τ*_*ves*_) with the vesicle relaxation time scale (*τ*_*rel*_), expressed by the equation^50^:

$${\tau }_{ves}\approx {\tau }_{rel}=\frac{R \eta }{\sigma}$$

where *R* is the equivalent sphere diameter of the vesicle, *η* is the viscosity and σ is the surface tension of the tektite melt. It follows that, by assigning to the tektite melt an average viscosity of ~ 10^4^ Pa s (calculated at 1 atm and 1400 °C using the viscosity model of Giordano et al.^51^) and a surface tension of 0.36 N/m (measured in a natural rhyolite at 1388 °C^52^) a characteristic relaxation time of 2.8 s is obtained for a vesicle of 100 µm diameter. The vesicle relaxation time scale increases proportionally with the vesicle size and, considering that the sphericity of the free vesicles decreases with their increasing size (Fig. 4a), it is possible to constrain the time scale for the formation of MN tektite in the order of seconds. This is in accordance with the maximum quench time of 10 s proposed by Walter^9^, that is required to prevent the full transformation of coesite into cristobalite at high temperature.

If the same consideration is applied to the vesicles constituting the froth layer of coesite-bearing inclusions, the generally smaller size and sphericity of the froth vesicles compared to free vesicles (Fig. 4b) testifies to a shorter time for the formation and quench of the froth structure, compared to what is inferred for the tektite melt. Hence, by considering an average size (*R*) of 10 µm for the highly deformed froth vesicles, the time scale for the formation of the froth layer could be estimated to be less than 1 s. This comparison suggests that coesite-bearing inclusions may have experienced a much more rapid quench compared to the tektite melt, likely due to energy dissipation during the froth expansion. This would represent a reasonable explanation of how the coesite-bearing frothy inclusions were preserved in the tektite melt.

### Coesite survival in impact melt rocks

The MN tektite sample MP26 lacks petrographic features that could testify to pressure exceeding 10 GPa, such as planar deformation features (PDF) in quartz or stishovite. On the counterpart, peak temperatures in excess of 1700 °C (melting point of pure SiO_2_) are indicated by the presence of lechatelierite inclusions. Upon shock unloading, at any temperature above the liquidus temperature of the tektite (~ 1130 °C; calculated at 0.1 GPa using Rhyolite-MELTS code^53^; Table S1), the back transformation of coesite into a more stable form of SiO_2_ would be expected to occur, making the retrieval of coesite in MP26 quite surprising. Glass et al.^10^ argued that the froth layer surrounding the coesite-bearing inclusion was determinant for the survival of coesite within the core of the inclusions, but the physical processes responsible of the froth formation were not investigated in detail. The new 3D textural characterization demonstrated that the froth layer encloses effectively the coesite-bearing inclusions (Fig. 3c; Fig. S4) and allowed to formulate new hypotheses on the formation mechanisms and time scales of the froth structures in MP26. In this frame, the role of the froth on the survival of coesite was twofold. On one hand, it contributed to rapidly subtract heat from the inclusion core through the endothermic process of gas exsolution and expansion, while, on the other hand, it provided thermal insulation to the core from the surrounding hot tektite melt. The preservation of the coesite was indeed possible because the unstable vesicle packing condition of the froth was created in a very short time (< 1 s) and maintained till the ultimate quench of the tektite melt to a glass (occurred in few seconds). These conditions did not occur for all the inclusions as, in some cases, the froth did not fully enclose the inclusion core or the froth vesicles coalesced and detached from the relict quartz surfaces. In these cases, the thermal insulation was not efficient and the inclusion underwent complete melting generating lechatelierite (Fig. 1g,h).

We show that the development of froth structures around mineral or lithic inclusions during the post-shock decompression was instrumental in preserving shock-produced coesite in a Muong Nong tektite. Note that the wide applicability of the present conclusion derived from detailed investigations on the Muang Phin tektite (sample MP26) is suggested by the observation of very similar coesite/quartz/froth relationships in another MN tektite specimen (sample MN20) collected 8 km North of Muong Nong village. The presence of small gaps in the relict quartz rim, corresponding to less vesiculated regions of the froth (Fig. 6a), as well as the increasing vesicle size outwards of the froth (Fig. 6b; Fig. S2a), confirm that the presence of surfaces for heterogeneous vesicle nucleation was fundamental for the development of the froth. This is further supported by the identification of coesite in frothy inclusions within an impact glass of Pantasma crater, Nicaragua^54^ (sample P20-3; Figs. 6c,d; Fig. S2b). Highly vesicular textures, froth and foam commonly occur in impact melt rocks, suevite and impact melt spherules from several impact structures, e.g., Barringer, Kamil, Bosumtwi, Ries, Rochechouart, Lonar, etc., and impact sites, e.g., Libyan Desert Glass field^55^. Decompression foamy structures may thus be important petrographic settings in which to search for key shock metamorphic mineral assemblages in impact melt rocks. This maybe particularly useful in addressing the nature of enigmatic natural glasses whose impact origin is still debated due to the lack of a high-pressure record like, for instance, the Dakhla and other similar glasses^56–59^.

## Tektite samples

The MN tektite sample MP26 is the same sample as that investigated by Glass et al.^10^, collected in the general area of Muang Phin, Laos (16° 32′ N Lat, 106° 01′ E Long) as reported by Schnetzler and McHone^60^. Two polished thin sections of MP26 were prepared for mineralogical and petrographic investigation by optical microscopy, Fourier transformed infrared spectroscopy (FTIR), and field emission gun-scanning electron microscopy (FEG-SEM). Three cylindrical cores with diameter of ~ 10 mm (MP26a), ~ 5 mm (MP26b) and ~ 3 mm (MP26c) and a height of 10 mm were obtained from a bulk piece of MP26 and analysed by a multiscale approach employing the X-ray Computed micro-Tomography technique with different experimental setups (Table 1). In particular, samples were imaged using a commercial 3D X-ray microscope system (XRM, MP26a), a custom-developed microfocus X-ray CT station (MCT, MP26a) and a synchrotron radiation-based set up (SR-µCT, MP26b and MP26c).

The second MN tektite briefly presented here (MN20) was collected by two of us (PR and SS) in January 2019 within a gravel quarry 8 km North of Muong Nong village (16° 26.543′ N Lat, 106° 28.825′ E Long). One lighter colored fragment with white inclusions and abundant vesicles similar to MP26 was selected among a total of 320 g of fragments, mounted in an epoxy disk and polished for FEG-SEM and Raman analyses (Fig. S2). Finally, a 1.3 g glass fragment (P20-3) was collected by two of us (PR and EN) in March 2020 within the Pantasma crater depression (13° 24.015′ N Lat, 85° 55.241′ W Long) was also mounted in an epoxy disk and polished for FEG-SEM and Raman analyses (Fig. S2).

### Analytical methods

Chemical analyses of major elements were carried out at the Centro per l'Integrazione della Strumentazione Scientifica (CISUP)—Università di Pisa (Italy), using a FEI Quanta 450 Field Emission Scanning Electron Microscope equipped with an EDX spectrometer Bruker QUANTAX XFlash Detector 6|10 (analyses reported in the supplementary material). FTIR spectra were acquired on a thin slab of MP26 double polished at a thickness of 285 μm. Several spectra were collected along a transect across the sample with a step size of 150 μm, a large spot size (250 × 250 μm^2^) was adopted in order to improve S/N ratio and average the effect of bubbles. FTIR spectra were acquired using a Bruker Hyperion 3000 microscope equipped with a KBr-broadband beam splitter, a 15X objective and a liquid nitrogen cooled mercury cadmium telluride detector at Laboratori Nazionali di Frascati-Istituto Nazionale di Fisica Nucleare (LNF-INFN, Frascati, Rome, Italy). The nominal resolution was set at 4 cm^−1^, and 64 scans were averaged for both spectrum and background.

### Lab-based CT measurements

The 3D characterization of the bulk tektite sample and of the coesite-bearing frothy inclusions was performed by high-resolution X-ray computed tomography (CT) using three different set ups. Sample MP26a has been analysed with the TomoLab station^61,62^, a X-ray microtomographic (MCT) system custom-developed at the Elettra synchrotron laboratory in Basovizza (Trieste, Italy) and based on a microfocus source (Hamamatsu, L9181, minimum focal spot size = 5 µm) allowing to work in a voltage range of 40–130 kV with a maximum power of 39 W. A water-cooled, 12-bit CCD camera (Photonic Science, VHR) has been used as detector. This camera has a 4008 × 2672 pixels full frame imager coupled by a fiber-optic tape to a gadolinium oxysulphide scintillator and an effective pixel size of 12.5 µm × 12.5 µm yielding a maximum field of view (FOV) of about 50 mm × 33 mm. The experimental parameters used for the tomographic scans are reported in Table S2. The tomographic reconstruction was performed using the commercial software COBRA 7 (Exxim, USA) based on the Feldkamp algorithm^63^. Volume visualization with volume rendering procedures (Fig. 3a) was performed by means of the commercial software VGStudio MAX 2.0 (Volume Graphics, Germany).

The same sample MP26a has been measured by using a Zeiss Xradia 620 Versa X-ray Microscope (XRM), adopting voxel resolutions of 12.48 µm, 4.24 µm, 1.092 µm and 0.998 µm. This instrument can operate in a voltage range of 30–160 kV with a maximum power of 25 W. The two stages magnification using scintillators screens coupled to a high-resolution microscope camera (2048 × 2048 pixels) allow Scout-and-Zoom workflows, where features and regions of interest can be identified during a short coarse resolution scan. Then, it is possible to zoom in by selecting a higher magnification objective to visualize structure at high resolution in the sample volume with a non-destructive method. For the current study, two overview scans with voxel sizes of 12.48 µm and 4.25 µm were performed before performing high-resolution scans at the microscale. The experimental conditions adopted for the different scans are reported in Table S2. The tomographic reconstruction was performed using the software integrated in the XRM instrument.

### Synchrotron radiation-based CT measurements

Samples M26b and MP26c have been measured by synchrotron radiation X-ray microtomography (SR-µCT) at the SYRMEP beamline of Elettra^64,65^ using a filtered polychromatic X-ray beam (filters = 1.5 mm Si + 1 mm Al). The detector consisted of a 16-bit, water-cooled, sCMOS macroscope camera (Hamamatsu C11440-22C) with a 2048 × 2048 pixels chip coupled through a high numerical aperture optics to a 17 µm-thick GGG:Eu scintillator screen. For sample MP26b the effective pixel size of the camera was set at 2.5 μm (FOV ~ 5.1 mm × 4.0 mm) while it was set at 0.9 µm (FOV ~ 1.8 mm × 1.8 mm) for sample MP26c. Scans were acquired in propagation-based phase-contrast mode^66^ setting a sample-to-detector distance of 150 mm. For each sample 900 (pixel size = 2.5 µm) or 1800 projections (pixel size = 0.9 µm) were acquired over a total scan angle of 180° and with an exposure time/projection of 2.5 s. For cores having a maximum height exceeding the vertical FOV of the camera, scans were acquired at different positions along the vertical axis then stitching the reconstructed volumes. Scans at 0.9 µm of pixel size were acquired in local area mode zooming in regions of interest of the sample. Volume visualization with volume rendering procedures (Fig. 3b,c) was performed by means of the free software Fiji^67^ using the 3D Viewer plugin.

## Supplementary information

Supplementary Information 1.

Supplementary Information 2.

Supplementary Video 1.

Supplementary Video 2.
