# Supplementary material for: 3D X-ray tomographic analysis reveals how coesite is preserved in Muong Nong-type tektites
Source: Sci Rep. 2020 Nov 26;10:20608. doi: 10.1038/s41598-020-76727-6 (PMC7691353; doi:10.1038/s41598-020-76727-6)
Supplement: Supplementary file 2 — Supplementary Information 2. [file 41598_2020_76727_MOESM2_ESM.pdf]

Supplementary material to:

3D tomographic analysis reveals how coesite is preserved in Muong Nong-type tektites

Matteo Masotta[1,2,*], Stefano Peres[1,3], Luigi Folco[1,2], Lucia Mancini[4,5], Pierre Rochette[6], Billy P. Glass[7], Fabrizio Campanale[1,2], Nicolas Gueninchault[9], Francesco Radica[10], Sounthone Singsoupho[11], Enrique Navarro[12]

[1] *Dipartimento di Scienze della Terra, Università di Pisa, Pisa, Italy*
[2] *CISUP, Centro per l'Integrazione della Strumentazione dell'Università di Pisa, Pisa, Italy*
[3] *Department of Lithospheric Research, University of Vienna, Wien, Austria*
[4] *Elettra - Sincrotrone Trieste SCpA, Basovizza, Trieste, Italy*
[5] *LINXS - Lund Institute for Advanced Neutron and X-ray Science, Lund, Sweden*
[6] *CEREGE, Aix-Marseille University, CNRS, INRA, IRD, Aix-en-Provence, France*
[7] *Department of Earth Sciences, University of Delaware, Newark, Delaware, USA*
[8] *Center for Nanotechnology Innovation@NEST, Istituto Italiano di Tecnologia (IIT), Pisa, Italy*
[9] *Zeiss Research Microscopy Solutions, Carl Zeiss SAS, Marly-le-Roi, France*
[10] *Dipartimento di Scienze, Università degli Studi Roma Tre, Roma, Italy*
[11] *Department of Physics, Faculty of Natural Sciences, National University of Laos, Vientiane, Laos*
[12] *Instituto di Geologia y Geofisica CIGEO, Universidad Nacional Autónoma de Nicaragua, Managua, Nicaragua*

*correspondence to matteo.masotta@unipi.it

FEG-SEM analyses and Rhyolite-MELTS simulations

Chemical analyses were performed on a polished thin section of MP26 using a FEI Quanta 450 Field Emission Scanning Electron Microscope equipped with an EDX spectrometer Bruker QUANTAX XFlash Detector 6|10 installed at the Centro per l'Integrazione della Strumentazione Scientifica (CISUP) - Università di Pisa (Italy). Chemical analyses were performed following traverses of 100 to 200 µm length with a spacing of 5 to 10 µm, in order to capture the chemical variation of the tektite layers, evidenced by different brightness in the backscattered electron images (Fig. S1). Chemical analyses are reported in Table S1 along with *liquidus* temperature (T_{liq}) calculated at 0.1 GPa using Rhyolite-MELTS code[1]. Rhyolite-MELTS is an updated version of MELTS, in which the enthalpies of formation of both quartz and endmember $KAlSi_3O_8$ in feldspar solid solution have been adjusted. Rhyolite-MELTS is to be preferred to MELTS for modelling phase relations in hydrous silicic systems.

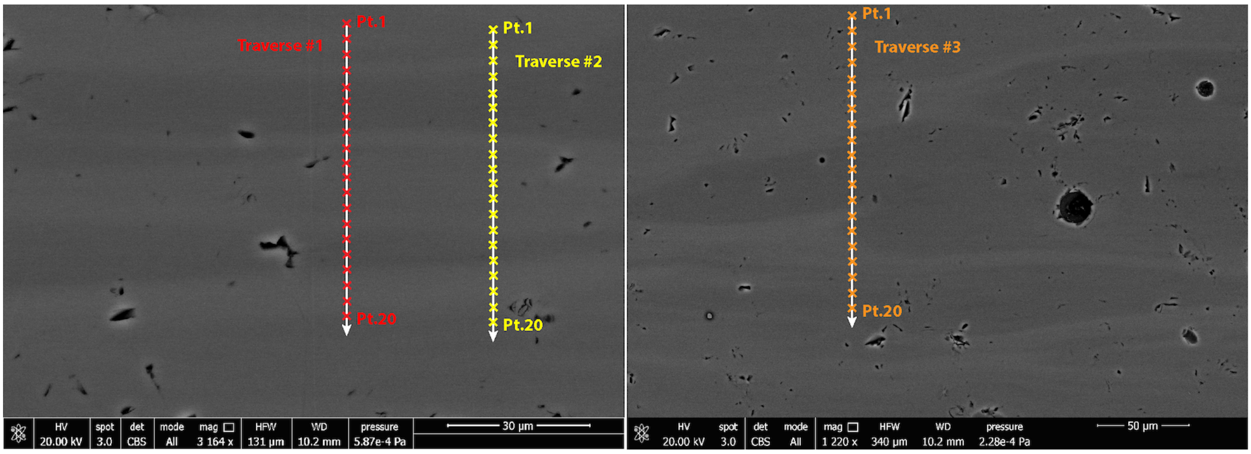

Figure S1. *Backscattered electron images of sample MP26 showing the location of the traverses.*

Table S1. Major oxide composition of the glass of MP26 (normalized to 100 wt.% on anhydrous basis) and *liquidus* temperatures (T_{liq}) calculated at 0.1 GPa using Rhyolite-MELTS code (only for traverse 1).

Pt #	SiO_2	TiO_2	Al_2O_3	FeO	MgO	CaO	Na_2O	K_2O	T_{liq} 0.1 GPa
Traverse 1 - 20 points - spacing 5 μm									
pt.1	73.41	0.69	12.51	4.51	1.84	3.23	1.03	2.79	1123
pt.2	71.15	0.83	14.61	4.73	2.46	2.48	1.64	2.10	1154
pt.3	72.94	0.87	13.24	3.65	2.17	2.91	1.42	2.82	1136
pt.4	72.93	0.55	12.55	4.65	1.65	3.28	1.29	3.11	1114
pt.5	75.01	0.83	12.53	3.36	1.54	2.42	1.54	2.79	1112
pt.6	75.63	0.99	11.80	3.85	1.33	2.53	0.99	2.88	1152
pt.7	74.99	0.63	11.61	4.59	1.40	2.86	0.98	2.96	1131
pt.8	71.76	1.22	12.98	5.23	1.70	3.15	1.18	2.78	1116
pt.9	73.19	0.73	12.82	4.74	1.99	2.87	1.07	2.61	1135
pt.10	72.06	1.05	12.15	5.32	2.01	3.14	1.23	3.05	1142
pt.11	72.89	0.97	13.30	4.77	1.64	2.71	1.31	2.42	1109
pt.12	73.05	1.05	12.92	4.47	1.75	2.88	1.26	2.63	1115
pt.13	74.24	1.02	12.02	4.26	1.65	2.62	1.20	3.00	1114
pt.14	75.50	0.64	11.86	3.42	1.49	2.90	1.21	3.00	1124
pt.15	71.35	1.00	13.04	5.03	2.11	3.64	0.88	2.96	1139
pt.16	69.56	1.12	13.94	5.12	2.38	3.80	1.39	2.70	1146
pt.17	71.67	0.64	14.03	4.52	2.02	2.96	1.36	2.81	1132
pt.18	75.12	0.58	12.13	4.59	1.32	2.51	1.15	2.61	1142
pt.19	72.38	0.86	12.41	5.32	1.93	2.91	1.04	3.17	1140
pt.20	73.85	0.84	12.48	4.50	1.66	2.88	1.20	2.60	1111
Traverse 2 - 20 points - spacing 5 μm									
pt.1	72.04	0.68	13.56	4.54	2.17	3.05	1.24	2.71	-
pt.2	73.99	0.88	12.22	4.58	1.89	2.66	1.17	2.62	-
pt.3	72.06	1.19	13.18	4.48	2.15	3.16	1.25	2.54	-
pt.4	73.13	0.96	12.46	4.68	1.79	2.91	1.30	2.79	-
pt.5	74.67	1.18	11.86	3.82	1.41	2.73	1.07	3.28	-
pt.6	74.43	1.11	12.23	4.17	1.63	2.44	1.14	2.87	-
pt.7	75.16	0.73	11.79	4.18	1.58	2.64	1.14	2.78	-
pt.8	71.55	0.92	13.93	4.41	2.19	3.10	1.32	2.59	-
pt.9	71.41	1.08	13.17	4.88	1.98	3.34	1.14	3.01	-
pt.10	71.71	1.04	13.65	3.95	2.22	3.10	1.46	2.87	-
pt.11	73.67	0.52	12.40	4.66	1.83	3.17	1.07	2.70	-
pt.12	72.23	1.05	13.03	4.79	1.97	2.99	1.18	2.75	-
pt.13	72.16	1.01	13.16	4.75	1.89	3.23	1.07	2.75	-
pt.14	73.09	1.00	12.63	4.49	1.89	2.93	1.23	2.75	-
pt.15	73.60	1.30	12.27	3.77	1.74	2.94	1.32	3.07	-
pt.16	71.11	0.98	13.41	5.20	2.11	3.38	1.16	2.68	-
pt.17	68.68	0.85	15.16	5.35	2.29	3.40	1.65	2.63	-
pt.18	71.72	0.80	12.82	5.00	2.10	3.45	1.18	2.93	-
pt.19	73.99	0.57	11.86	4.86	1.78	2.94	1.01	3.01	-
pt.20	74.48	1.01	11.88	4.37	1.62	2.67	1.27	2.71	-
Traverse 3 - 20 points - spacing 10 μm									
pt.1	72.77	0.67	12.79	4.15	1.97	3.21	1.56	2.88	-
pt.2	72.65	1.00	12.26	5.06	1.70	3.24	1.27	2.82	-
pt.3	72.47	0.84	12.94	4.61	2.05	3.08	1.39	2.63	-
pt.4	73.77	0.61	12.63	4.19	1.95	3.15	1.27	2.45	-
pt.5	73.47	0.84	12.37	4.36	1.90	3.23	1.18	2.65	-
pt.6	72.13	0.64	12.85	4.73	1.92	3.32	1.47	2.95	-
pt.7	70.18	0.99	13.77	4.88	2.39	3.56	1.62	2.64	-
pt.8	70.52	0.94	13.99	4.49	2.27	3.49	1.41	2.89	-
pt.9	71.44	1.11	13.03	4.91	2.14	3.34	1.27	2.78	-
pt.10	75.43	0.88	11.97	3.47	1.50	2.56	1.35	2.85	-
pt.11	74.96	0.92	12.24	3.65	1.69	2.36	1.35	2.84	-
pt.12	74.43	0.89	12.75	3.57	1.81	2.28	1.42	2.86	-
pt.13	75.70	1.01	12.00	3.45	1.37	2.43	1.23	2.82	-
pt.14	73.15	0.68	13.30	4.32	1.85	2.58	1.53	2.61	-
pt.15	69.25	0.87	14.64	5.83	2.46	3.29	1.23	2.45	-
pt.16	70.40	0.78	14.41	4.95	2.29	3.21	1.30	2.66	-
pt.17	70.98	1.09	13.10	5.59	2.11	3.27	1.16	2.71	-
pt.18	73.50	0.71	12.01	4.90	1.84	2.93	1.37	2.75	-
pt.19	72.35	1.13	12.35	4.79	1.84	3.30	0.99	3.26	-
pt.20	74.70	1.02	11.97	4.09	1.34	2.59	1.18	3.12	-

Raman analyses of MN20 and P20

Raman spectra of SiO_2 rich inclusions in two polished sections of samples MN20 and P20 (Fig. S2) were obtained in Lyon using a LabRAM HR800 Evolution spectrometer that has a confocal Czerny-Turner geometry and a laser source of 532 nm in wavelength. Each spectrum was acquired with a power of 10 mW, and 25 accumulations of 5 to 15 s. Gratings with 600 groove/mm were used in order to cover the frequency range 60 to 1300 cm^{-1}.

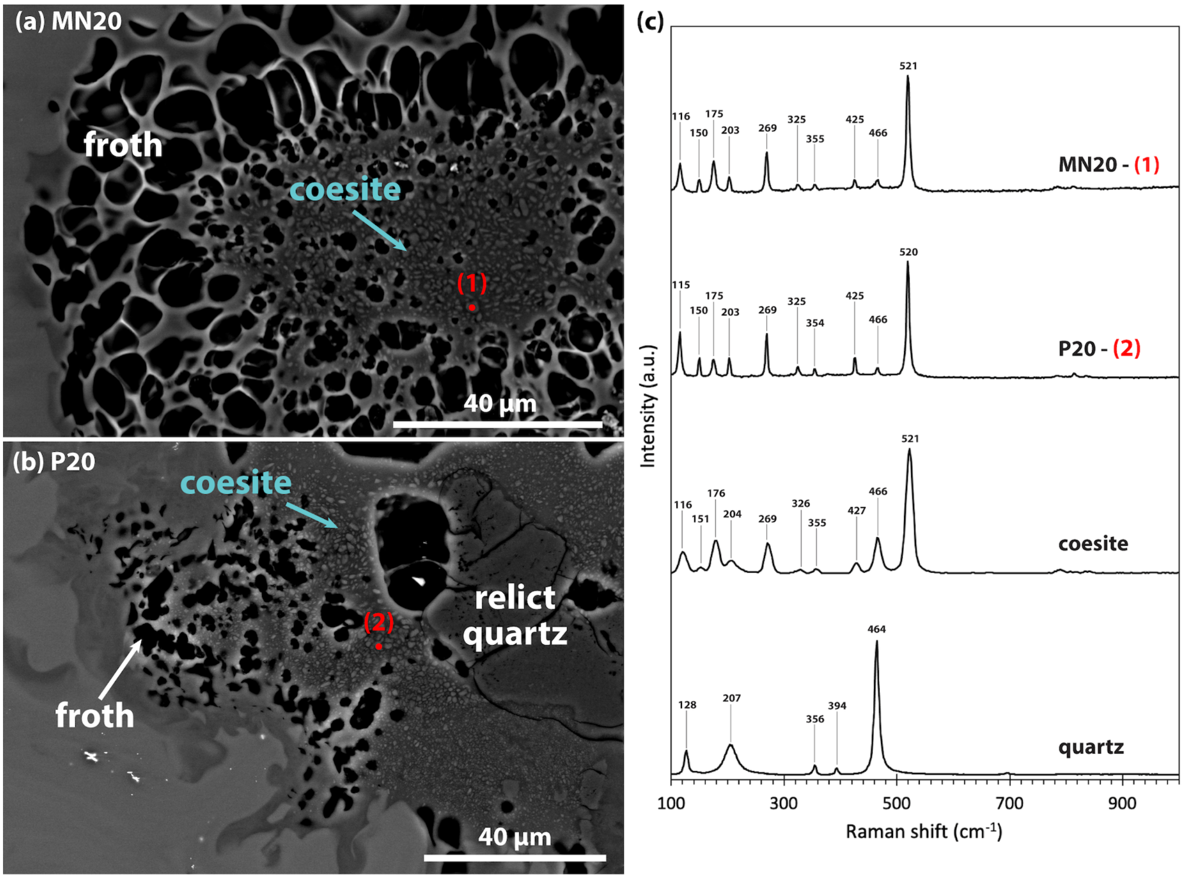

Figure S2. *Backscattered electron images of samples MN20 (a) and P20 (b) indicating the spot location of Raman analyses (c). The Raman spectra of coesite and quartz are reference spectra from RRUFF database.*

Analysis of SR-µCT tomographic data

Reconstruction of SR-µCT tomographic data. The experimental parameters used for the tomographic scans are reported in Table S2. The reconstruction of the tomographic data was carried out by using the *SYRMEP Tomo Project (STP)* software suite[2], applying pre-reconstruction filters for reducing ring artefacts caused by detector inhomogeneity. Prior to reconstruction, a single-distance phase-retrieval procedure[3] was applied to sample projections. This procedure allows to improve both the reliability of the further segmentation process, and related morphological and textural analyses, and to fully exploit the potential of phase-contrast imaging. Combining phase-retrieval with the Filtered Back-Projection algorithm[4] it is possible to obtain the 3D distribution of the refraction index of samples with constant composition, and thus characterized by a constant ratio $\gamma = \delta/\beta$ between the real and imaginary parts of the refractive index at a given X-ray energy. It was demonstrated that this kind of algorithm can also be employed on multiphase rock samples imaged with a filtered polychromatic synchrotron X-ray beam[5]. In order to enhance the contrast between vesicles and rock matrix the γ ratio was set at 50 and 29 for the 2.5 µm and 0.9 µm settings, respectively.

Table S2. Experimental conditions adopted for lab-based X-ray microtomographic scans

Sample	Method	Voltage /Power (V/W)	Effective pixel size (µm)	Bin	Number of projections over 360°	Exp. time/ projection (sec)	Total scan time (h)	Source-sample distance (mm)	Source-detector distance (mm)
MP26 (10mm)	XRM	80/10	12.480	2x2	2600	12.78	3.0	58.02	254.58
MP26 (10mm)	XRM	80.10	4.245	1x1	3800	8.69	18.7	26.00	180.00
MP26 (10mm)	XRM	100/14	1.092	2x2	6400	1.01	9.4	9.54	55.40
MP26 (10mm)	XRM	100/14	0.998	2x2	6400	1.01	9.4	10.61	55.40
MP26 (10mm)	MCT	130/8	5.000	2x2	2400	6.00	4.1	80.0	320.00

Legend: XRM: X-ray microscopy, MCT: microfocus X-ray tomography, Bin: pixel binning of the detector.

3D image processing and analysis. The 3D image processing and analysis of MCT and SR-µCT data was performed using the *Pore3D* software library developed at Elettra[6-7] which allows extraction of quantitative microstructural and textural parameters of porous and multiphase systems. Segmentation and analysis of XRM data was performed using the free software *Fiji*[8] (Version 2.0.0-rc-69/1.52p). Examples of pore and inclusion segmentation are reported in Figs. S3 and S4, while results of the image analysis are reported in the supplementary Excel spreadsheets. The tools available in *Pore3D* and *Fiji* allowed a quantitative description of the morphology and topology of the components (vesicles and coesite-bearing inclusions), as well as the analysis of the bulk porosity. In order to perform the textural analysis, several volumes of interest (VOIs) were extracted from each sample (Table 1). Only VOIs of the order of or larger than 60 mm^3 can be considered representative of the grain heterogeneities and thus defined as Representative Elementary Volume (REV). After the extraction of the VOIs, XRM and SR-µCT data were filtered in order to remove noise and enhance edges by means of a 3D Bilateral Filter, which smooths images but preserves edges applying a non-linear combination of nearby image grey-level values. Image segmentation to obtain binary volumes containing only the objects of interest was performed by a manual 3D Otsu algorithm[9] on all volumes containing groundmass, vesicles and coesite-bearing inclusions. Outliers were then removed from the segmented images corresponding to the cut-off values listed in Table 1.

The *Pore3D Basic Analysis module*[6] was used to compute the vesicularity parameter (i.e., the fraction of vesicles in the selected VOI) and the topological characteristics, as described by the Minkowski functionals[10-11] such as: specific surface area (surface area of all vesicles divided by the total VOI), integral of mean curvature (index of the dominance of convex or concave shapes[12], and Euler characteristic (index of connectivity of the object network[13-14]. The results of the Basic analysis are reported in Table S3. In particular, the Euler characteristic has the attributes of a topological order parameter describing the spatial connectivity where positive values typically consist of isolated objects dispersed in the matrix. Samples feature also positive values of the integral of the mean curvature, indicating a high fraction of convex surfaces[12,15], as occurs for samples dominated by isolated spherical voids.

After this computation, a filter was applied to the selected VOI in order to suppress blobs (connected components) connected to the image borders[6]. Then the *Blob Analysis module* of *Pore3D* was used to perform the analysis of each vesicle. This approach is based on the concept of maximal inscribed spheres. We then calculated the number of vesicles, their volume, the sphericity (ratio of the surface area of an equivalent sphere to the surface area of the object), aspect ratio (the ratio of the minimum and maximum axis of each vesicle) and diameter of the maximal inscribed sphere. The results of the Blob Analysis are also summarized in Table 1.

The 3D rendering of XRM data was done using the 3D Viewer plugin of *Fiji* while the volume (as reconstructed) and isosurface (segmented) renderings of MCT and SR-mCT data were obtained using both the commercial software *VGStudio Max 2.0* (Volume Graphics, Germany) and the 3D Viewer plugin of *Fiji*.

Table S3. Results of the *Pore3D Basic analysis* module for MCT and SR-μCT datasets

Sample	Method	Isotropic voxel size (μm)	VOI size (voxels)	VOI size (mm^3)	Vesicularity	Specific Surface Area (mm^{-1})	Integral of Mean Curv. (mm^{-2})	Euler characteristic (mm^{-3})
MP26	SR-μCT	2.50	1180x1200x920	2.95x3.0x2.3	0.088	5.05	372	4300
MP26	MCT	5.00	500x500x500	2.5x2.5x2.5	0.067	3.12	77	246
MP26	MCT	5.00	1000x1000x1000	5.0x5.0x5.0	0.049	2.56	71	261
MP26	MCT	5.00	1200x1200x1200	6.0x6.0x6.0	0.049	2.52	70	265

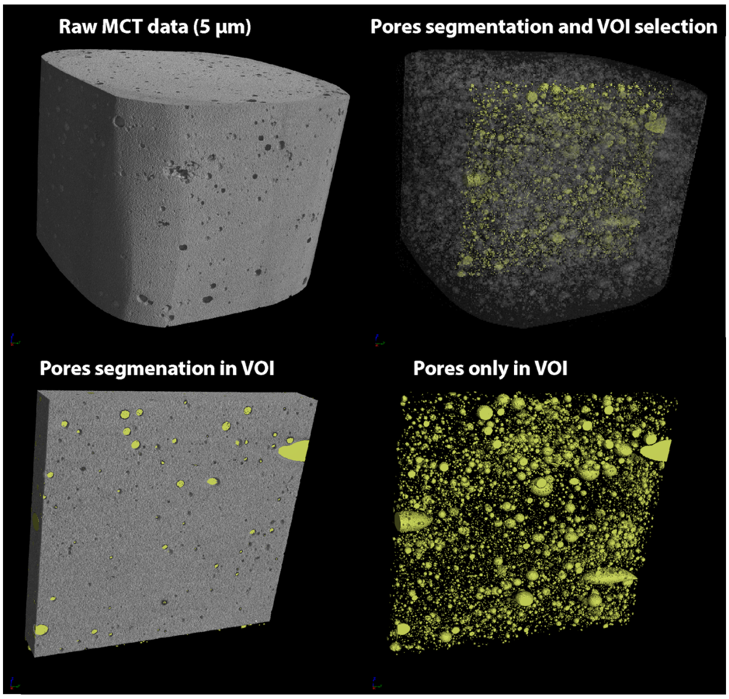

Figure S3. *Example of pores segmentation and extraction of a VOI from the MCT data on MP26a.*

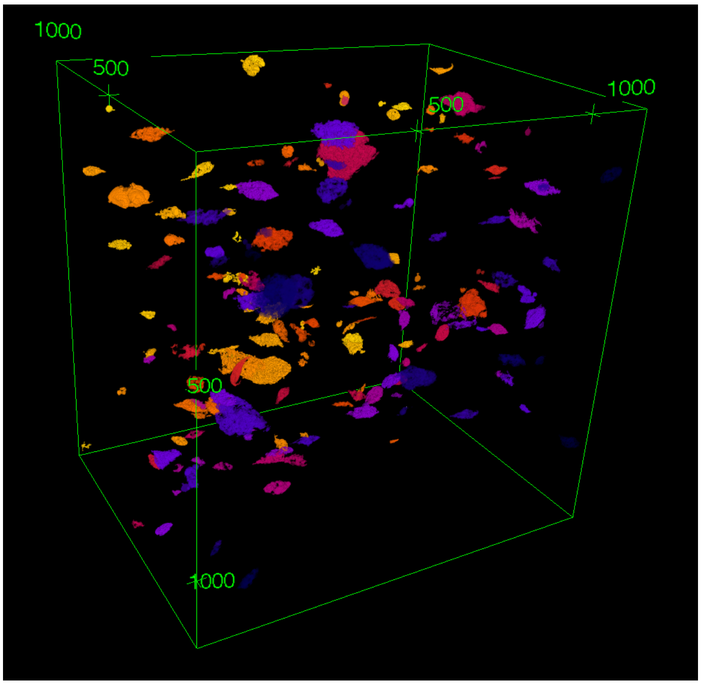

Figure S4. *Example of inclusions segmentation in a VOI from SR-μCT data on MP26b.*

References

1. Gualda, G.A.R., Ghiorso, M.S., Lemons, R.V. & Carley, T.L. Rhyolite-MELTS: A modified calibration of MELTS optimized for silica-rich, fluid-bearing magmatic systems. *J. Petrol.* **53**, 875-890 (2012).
2. Brun, F. *et al.* Enhanced and Flexible Software Tools for X-ray Computed Tomography at the Italian Synchrotron Radiation Facility Elettra. *Fundam. Inform.* **141**, 233–243 (2015).
3. Paganin, D., Mayo, S.C., Gureyev, T.E., Miller, P.R. & Wilkins, S.W. Simultaneous phase and amplitude extraction from a single defocused image of a homogeneous object. *J. Microsc.* **206**, 33–40 (2002).
4. Herman, G.T. Image Reconstruction from Projections: The Fundamentals of Computerized Tomography. Academic Press, New York 1980.
5. Arzilli, F. *et al.* Near-liquidus growth of feldspar spherulites in trachytic melts: 3D morphologies and implications in crystallization mechanisms. *Lithos* **216-217**, 93–105 (2015).
6. Brun, F. *et al.* Pore3D: A software library for quantitative analysis of porous media. *Nucl. Instrum. Meth. A* **615**, 326–332 (2010).
7. Zandomeneghi, D. *et al.* Quantitative analysis of X-ray microtomography images of geomaterials: Application to volcanic rocks. *Geosphere* **6**, 793–804 (2010).
8. Schindelin, J. et al. Fiji: an open-source platform for biological-image analysis. *Nat. Methods* **9**, 676–682 (2012).
9. Otsu, N. A threshold selection method from gray level histograms. *IEEE T. Syst. Man Cybern.* **9**, 62–66 (1979).
10. Steele, J.H. Characterizing 3D microstructure using the Minkowski Functionals. *Microsc. Microanal.* **13**, 1658–1659 (2007).
11. Ohser, J. & Schladitz, K. 3D images of material structures: Processing and analysis: Weinheim, Germany, Wiley-VCH (2009).
12. Russ, J.C. & DeHoff, R.T., Practical Stereology. (New York, Plenum Press, 1986).
13. Odgaard, A. & Gundersen, H.I.G. Quantification of connectivity in cancellous bone, with special emphasis on 3D reconstructions. *Bone* **14**, 173–182 (1993).
14. Ohser, J. & Mücklich, F. Statistical Analysis of Microstructure in Material Science. In Barnett, V., ed., Statistics in Practice: West Sussex, England, John Wiley & Sons, 381 (2000).
15. Velichko, A., Holzapfel, C., Siefers, A., Schladitz, K. & Mücklich, F. Unambiguous classification of complex microstructures by their three-dimensional parameters applied to graphite in cast iron. *Acta Mater*. **56**, 1981–1990 (2008).
